# The Impact of Advanced Maternal Age on Pregnancy Outcomes: A Retrospective Multicenter Study

**DOI:** 10.3390/jcm12175696

**Published:** 2023-09-01

**Authors:** Hila Hochler, Michal Lipschuetz, Yael Suissa-Cohen, Ari Weiss, Hen Y. Sela, Simcha Yagel, Joshua I. Rosenbloom, Sorina Grisaru-Granovsky, Misgav Rottenstreich

**Affiliations:** 1 Department of Obstetrics and Gynecology, Hadassah Medical Center Mount-Scopus, Faculty of Medicine, Hebrew University of Jerusalem, Jerusalem 9765422, Israel; 2 Henrietta Szold School of Nursing, Faculty of Medicine, Hadassah and the Hebrew University, Jerusalem 91120, Israel; 3 Department of Obstetrics & Gynecology, Shaare Zedek Medical Center, Faculty of Medicine, Affiliated with the Hebrew University School of Medicine, Jerusalem 91031, Israelsorina@szmc.org.il (S.G.-G.); misgavr@gmail.com (M.R.); 4 Department of Obstetrics and Gynecology, Hadassah Medical Center Ein-Kerem, Faculty of Medicine, Hebrew University of Jerusalem, Jerusalem 91121, Israel; 5 Department of Nursing, Jerusalem College of Technology, Jerusalem 9548301, Israel

**Keywords:** advanced maternal age, perinatal outcomes, cesarean delivery, preterm delivery, NICU admission, neonatal asphyxia, mechanical ventilation

## Abstract

The aim of this multicenter retrospective cohort study was to examine the impact of maternal age on perinatal outcomes in multiparas, stratified according to maternal age in one- and two-year increments. The analysis involved 302,484 multiparas who delivered between the years 2003 and 2021 in four university-affiliated obstetrics departments. Maternal age was considered both as a continuous variable and in two-year intervals, as compared with a comparison group of parturients aged 25–30 years. The study focused on cesarean delivery and neonatal intensive care unit (NICU) admission as primary outcomes. The findings revealed that cesarean delivery rates increased as maternal age advanced, with rates ranging from 6.7% among 25–30 year olds, rising continuously from 13.5% to 19.9% between the age strata of 31 and 42, to exceeding 20% among those aged ≥ 43 years (*p* < 0.01 for each stratum when compared to 25–30 year old group). Similarly, NICU admission rates rose from 2.7% in the comparison group to 6% in parturients aged 45–46 years (*p* < 0.01 for each stratum when compared to 25–30 year old group). The study highlights the association between incrementally advanced maternal age and increased rates of maternal and neonatal complications, necessitating global awareness of these implications for family planning decisions and maternal care.

## 1. Introduction

In the course of recent decades, age at childbearing has trended older among women in developed countries, for social and economic reasons. The birth rate among older mothers has risen considerably in the last 40 years: among women aged 35–39 by 272% (to 54.2 births per 1000 women in 2021) and for women aged 40–44 years by 318% (to 12.1 births per 1000 women in 2021) [1,2]. This growing trend impacts individuals and health systems and should be considered in patient counseling and public health planning.

Advanced maternal age (AMA) has been shown to be associated with sub-optimal obstetric outcomes [3,4,5,6,7,8,9,10]. However, studies examining perinatal outcomes have suffered from methodological drawbacks, including a lack of stratification according to nulliparity vs. multiparity [3,4,6,7,8,9,10,11], the inclusion of multiple pregnancies [3], a lack of agreement on the threshold age, or wide maternal age increments [3,4,5,8]. Moreover, crucial issues are still subject to debate due to conflicting results in previous studies, e.g., the possible associations between maternal age and preterm birth [4,7,9,10] and NICU admission [4,6].

Previous investigations into the effects of AMA imposed a maternal age threshold, usually 35 or 40 years. As a result, most guidelines and clinicians refer to these ages when counseling women, despite their arbitrary selection. A few studies grouped maternal age into 5-year increments [3,4,5,7,8,9,10,11], and most reported that the effects of increasing age occurred along a continuum, rather than as a threshold. We found only one study in the literature that analyzed maternal age as a continuous variable (in addition to 5-year increments) [10], but the researchers did not propose conclusions for the continuous data analysis.

In this study, we aimed to analyze the perinatal outcomes of multiparous women with singleton pregnancies according to maternal age, in one- and two-year increments, in order to substantiate the notion of an inflection point after which outcomes worsen significantly. We focused on multiparous parturients, to control for the known detrimental effects of primiparity.

## 2. Methods

This was a retrospective, electronic health record-based (EHR), multicenter study performed on data from four university-affiliated obstetric departments in Jerusalem, Israel, encompassing the years 2003–2021. The Shaare Zedek Medical Center, Bikur Holim Medical Center, Hadassah Ein Kerem Medical Center, and Hadassah Mt. Scopus Medical Center all serve Jerusalem’s population (>1,200,000 patients). Together, these medical centers account for approximately 25% of all deliveries in Israel. Our cohort was drawn from diverse, multi-ethnic patient populations.

Our study utilized a retrospective design, encompassing all multiparous women aged 25–46 years who presented to our delivery wards with singleton live fetuses in vertex presentation, at or above 24 weeks of gestation. The inclusion criteria were applied consistently to all eligible individuals within the specified age range, ensuring a comprehensive representation of this specific population. We excluded primiparous women, multifetal gestation, pre-labor fetal death, non-vertex presentation, unknown maternal age, known major fetal malformation, known genetic abnormalities, and women who delivered out of hospital.

Furthermore, to minimize potential biases, we took specific steps to ensure blinding during the data collection process. Research staff members who were independent of any involvement in perinatal care conducted the data collection in a blinded fashion. This approach aimed to prevent any knowledge or bias related to the participants’ outcomes from influencing the data collection process. Additionally, all collected information was anonymized and de-identified before analysis, further safeguarding the integrity and confidentiality of the data.

The electronic health records were continuously updated in real time during labor, delivery, and surgery by attending healthcare professionals. In addition, the data were audited periodically by trained technical personnel to ensure their validity; as such, the possibility of bias inherent to retrospective studies was minimized.

For the purpose of the study, to delineate the effect of advanced maternal age (AMA) on maternal and neonatal outcomes, we compared outcomes among six two-year maternal age subgroups of multiparous parturients: 35–36, 37–38, 39–40, 41–42, 43–44, 45–46 years. These groups were compared to a comparison group of parturients aged 25–30 years.

The primary maternal and fetal outcomes considered were cesarean delivery (CD) and neonatal intensive care unit (NICU) admission, respectively.

The secondary outcomes were preterm delivery (PTD) at <37 0/7, <34 0/7, <32 0/7, and <28 0/7 weeks’ gestation.

Other maternal and neonatal adverse outcomes included premature rupture of membrane (PROM), retained placenta/placental fragments, severe perineal tear grade 3/4, shoulder dystocia, placental abruption, postpartum hemorrhage, blood product transfusion, maternal intensive care unit (ICU) admission, prolonged hospitalization (≥7 days after cesarean; ≥5 days after vaginal delivery), chorioamnionitis, puerperal fever, uterine rupture, explorative laparotomy, and hysterectomy; intrapartum intra-uterine fetal death (IUFD), 5 min Apgar score <7, small for gestational age (SGA), large for gestational age (LGA), congenital malformations, neonatal asphyxia, meconium aspiration, jaundice, transient tachypnea of the newborn, brachial plexus injury, mechanical ventilation, convulsions, hypoglycemia, sepsis encephalopathy, and intracranial hemorrhage (ICH). Maternal medical history was also examined, including previous miscarriages, fertility treatments, hypertensive disorders of pregnancy, pre-gestational and gestational diabetes, obesity (BMI > 30), smoking, and anemia.

Chorioamnionitis was defined by the presence of maternal fever (≥37.8 °C or ≥38.0 °C) plus two or more of the five following clinical signs: maternal tachycardia (heart rate >100 beats/min), fetal tachycardia (heart rate >160 beats/min), uterine tenderness, purulent or foul-smelling amniotic fluid or vaginal discharge, and maternal leukocytosis (white blood cell count >15,000/mm^3^) [12,13,14,15].

Puerperal fever was defined as a temperature of 38 °C/100.4 °F or higher on two measurements, exclusive of the first 24 h postoperatively [16]. Perinatal death was a dichotomous variable for any death from delivery until hospital discharge.

Birth weight categories were defined as SGA if birth weight was below the 10th percentile for gestational age and LGA was defined as birth weight above the 90th percentile for gestational age, with the use of customized population-based liveborn infant birthweight curves in Israel [17].

Neonatal asphyxia was defined as umbilical artery cord blood pH < 7.1. Encephalopathy was defined as Sarnat stages >2 [18].

The study was approved by the Institutional Review Boards of the Shaare Zedek Medical Center and Bikur Holim Hospital (IRB approval number: 110-22) and Hadassah (IRB approval number: 632-15). Data were obtained from medical records and de-identified, with no direct participation of patients. As such, written informed consent was not required.

### Statistical Methods

The characteristics were described as proportions (nominal variables), means ± SD (continuous variables with normal distribution), and medians with interquartile ranges (IRQ) (continuous variables without normal distribution), as appropriate. Categorical variables were compared using the Chi-square or Fisher’s exact tests and continuous variables were analyzed using the unpaired Student’s *t*-test, as appropriate. We performed a univariate analysis in which we compared the outcomes of women categorized by maternal age group. A *p* value <0.05 was considered statistically significant.

Variables found to be significant on univariate analysis (not presented) for the primary outcomes were included in multinomial multivariable logistic regression modeling of the association between maternal age and mode of delivery. These included the number of previous CDs, hypertensive disorders of pregnancy, assisted reproduction treatments, diabetes, previous miscarriages, gravidity, and parity.

Variables found to be significant on univariate analysis (not presented) for the primary outcomes were included in an additional two separate multivariable logistic regression models of the association between maternal age and preterm delivery (<37 0/7 weeks) and NICU admissions, including the number of previous CDs, hypertensive disorders of pregnancy, smoking, fertility treatments, diabetes, previous miscarriages, obesity (BMI > 30), gravidity, and parity.

The results of these analyses are reported as adjusted odds ratios (aOR) with 95% confidence intervals (CIs). All statistical tests were 2-sided. Analyses were carried out using the SPSS software (version 25 statistical package: IBM, Armonk, NY, USA).

## 3. Results

During the study period, we identified 419,465 deliveries in our medical centers; among them, 302,484 (72.1%) multiparous women met the inclusion and exclusion criteria. Of these, 74,032 (24.5%) women were 35 years old or above and were further stratified into six 2-year subgroups: 35–36 (27,089 patients), 37–38 (20,450 patients), 39–40 (13,928 patients), 41–42 (7802 patients), 43–44 (3396 patients), and 45–46 years old (y.o.) (1031 patients). Each subgroup was compared to 114,918 (38%) women aged 25–30 years, which constituted the comparison group. Additionally, there were 68,794 parturients aged 31–34 years, 114,918 women aged 25–30, and 44,740 women aged <24 years (Supplementary Figure S1).

Maternal demographic and obstetric characteristics comparing the study and comparison groups are presented in Table 1. The rate of the following increased significantly with increasing maternal age for all strata, compared with the comparison group (*p* < 0.01): fertility treatments, previous miscarriages, diabetes (pre-gestational and gestational), hypertensive disorders of pregnancy, and obesity (BMI ≥ 30). Rates of anemia at admission to labor (hemoglobin < 11gr%), as well as epidural analgesia use, decreased as maternal age rose, starting from the 37–38 y.o. subgroup. Rates of smoking and induction of labor were significantly higher in all maternal age subgroups above 35 years, but no continuous trend was demonstrated with rising maternal age beyond 35 years.

### 3.1. Primary Outcome—Maternal

**Figure 1:**
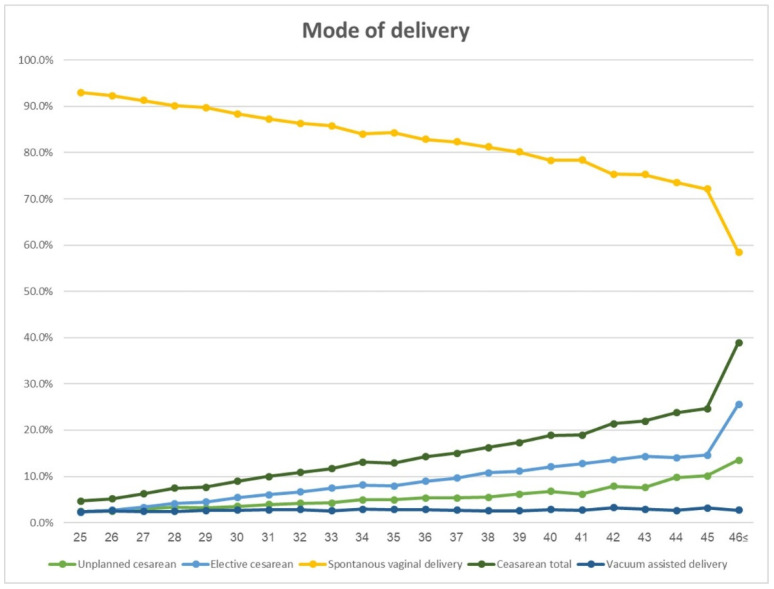
Mode of delivery by maternal age (years).

Obstetric and maternal delivery outcomes, stratified by maternal age subgroups, are presented in Table 2. Rates of planned, unplanned, and all cesarean deliveries increased as maternal age rose, from 6.7% among 25–30 y.o., rising continuously from 13.5% to 19.9% between the age strata of 31 and 42, and exceeding 20% among the strata 43 and above. Vacuum-assisted delivery remained steady across the maternal age strata. The association between the different modes of delivery and maternal age in single-year increments is presented in Figure 1.

### 3.2. Secondary Outcomes—Maternal

**Figure 2:**
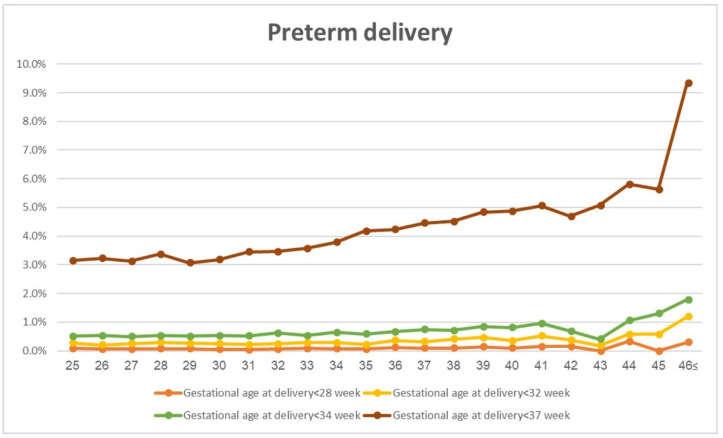
Preterm delivery by maternal age (years).

Rates of PTD (gestational age < 37 weeks and <34 weeks) increased with maternal age, from 3.2% PTD < 37 weeks in the comparison group, gradually rising to 6.1% in the 45–46 y.o. stratum; PTD < 34 weeks increased from 0.5% to 1.4% (Table 2). The vast majority of preterm deliveries prior to 37 weeks occurred at 34–37 weeks. The association between PTD and maternal age according to maternal age stratum is presented in Figure 2.

Rates of the following outcomes increased as maternal age increased: placental abruption, postpartum hemorrhage (PPH), blood product transfusion, puerperal fever, and prolonged hospital stay. Rates of hysterectomy were significantly higher in all maternal age subgroups above 35 years, but no continuous trend was demonstrated according to the maternal age subgroups. Rates of episiotomy and OASIS (grade 3/4) were lower in maternal age subgroups above 35 years.

### 3.3. Primary Outcome—Neonatal

**Figure 3:**
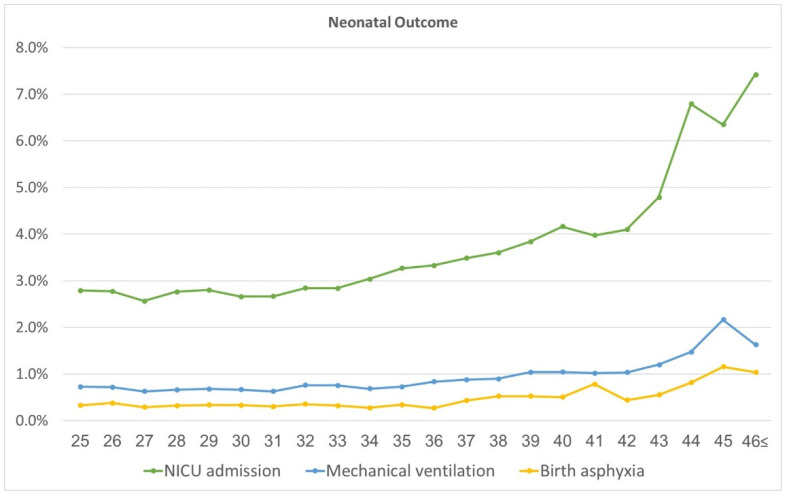
Neonatal outcomes by maternal age (years).

Neonatal outcomes, stratified by maternal age subgroups, are presented in Table 3. Rates of NICU admissions increased with the maternal age subgroup, from 2.7% in the control group, rising gradually according to maternal age to a peak of 6% among parturients aged 45–46 years. The progressive increase in NICU admissions, birth asphyxia, and neonatal mechanical ventilation, in single-year increments, is presented in Figure 3.

### 3.4. Secondary Outcomes—Neonatal

Rates of the following outcomes rose as maternal age increased: 5-min Apgar score < 7, mechanical ventilation, birth asphyxia, transient tachypnea of the newborn (TTN), hypoglycemia, and neonatal jaundice (Table 3, Figure 3). Neonatal birthweight and the rate of LGA increased with maternal age, up to 43–44 years. Notably, the rate of SGA also increased as the maternal age stratum increased.

Several adjusted multivariable logistic regression analyses for covariates and confounders were applied in order to examine the association between maternal age and perinatal outcomes (Table 4).

Multivariate modeling of maternal age and unplanned CD revealed a per-unit increase of 1.11 (1.10–1.11) for every added maternal year, with an aOR of 2.0 (95% CI 2.12–2.48) for unplanned CD in the 35–36 stratum, rising progressively to an aOR of 8.11 (95% CI 6.25–10.53) among women aged 45–46 years.

Multivariate modeling of maternal age and NICU admission, analyzing maternal age as a continuous variable, revealed a per-unit increase of 1.03 (1.01–1.04) for every added maternal year, while analyzing in two-year increments demonstrated an aOR of 1.2 (1.04–1.42) for NICU admission in the 35–36 stratum, rising progressively to an aOR of 1.9 (1.17–3.1) among women 45–46 years old.

Additional multivariate analysis for the association between maternal age and adverse neonatal outcome revealed a per-unit increase of 1.02 (1.02–1.03) for every added maternal year. Analysis in two-year increments demonstrated an aOR of 1.1 (0.99–1.2) in the 35–36 stratum, rising progressively to an aOR of 1.5 (1.07–2.05) among 45–46 y.o. women.

The same manner of association was found in models of maternal age and PTD < 37, elective CD, and vacuum extraction delivery. These analyses also revealed that maternal age was independently associated with these outcomes.

## 4. Discussion

This large multicenter study evaluated the maternal and neonatal outcomes of multiparous women with singleton pregnancies from age 35, in increments of one and two years. We showed a continuous increase in rates of maternal and neonatal complications with each year increase in maternal age, including CD, PTD, NICU admission, neonatal asphyxia, mechanical ventilation, SGA, TTN, hypoglycemia, and neonatal jaundice. Our data show that there is no maternal age threshold or inflection point after 35 years; rather, there is a gradual maternal age-associated increase for most maternal and neonatal adverse outcomes.

Our results indicate that among multiparas, after adjusting for potential confounders, the rates of both unplanned and planned CDs increase gradually as maternal age increases. This concurs in part with what has been shown in the literature [3,4,5,6,7,8,9,10,11,12]. Khalil and her associates [10] demonstrated an increase in the rate of elective and urgent CDs in univariate analysis. However, after adjusting for confounders, the increase in unplanned CDs associated with maternal age was not statistically significant, while an increased rate of planned cesarean remained significant. Other previous studies failed to differentiate between planned and unplanned CDs or perform logistic regression to control for confounders [11], in addition to the high rate of nulliparas, which are at an a priori increased risk of cesarean delivery. Our design excluded the fundamentally different population of primiparas to focus on multiparas and controlled for other potential confounders, showing significantly increasing rates of planned and unplanned CDs for each year increase in maternal age.

The current study also indicates an increase in NICU admissions as maternal age rises. NICU admission includes neonates born prematurely, as well as those born with a medical problem, making it a more comprehensive parameter to characterize neonatal condition at birth compared to PTD alone. Previous studies have shown conflicting results regarding this issue [3,4,6]. The increase appears to derive from the increased risk of PTD and comorbidities as maternal age rises.

Our data revealed an increased rate of PTDs before 37, 34, and 32 weeks as a function of maternal age, but not in deliveries <28 weeks. PTD < 37 and 32 weeks gradually increased to a peak of two-fold compared to the control group, and deliveries <34 weeks increased up to three-fold. Even after controlling for confounders, maternal age remained an independent factor for PTD. Most previous studies found an increase in the incidence of PTD at an older maternal age [3,9,19,20], although others did not demonstrate this phenomenon^4^. Importantly, although most of the rise in PTD in our cohort was explained by births under 34 weeks, and there was no increase in births under 28 weeks, we observed a statistically significant increase in adverse neonatal outcomes.

The pathophysiologic explanation for the increase in PTD with maternal age includes an increase in iatrogenic preterm births due to underlying diseases such as hypertension, as well as dysfunction of the placenta and the maternal heart, which causes an increase in the incidence of preeclampsia and fetal growth restriction (FGR). It is possible that there are additional mechanisms related to aging involved in this process that remain to be elucidated.

The increased rate of diabetes and hypertensive disorders associated with advancing maternal age in our cohort concurs with previous studies [3,4,5,6,7,8,9,10,11,20].

An important finding in our data is an increase in severe complications of the newborn associated with rising maternal age: asphyxia, the need for mechanical ventilation, SGA, TTN, hypoglycemia, and jaundice of the newborn. We found one study that investigated a wide range of neonatal complications according to maternal age. Gilbert and his associates [20] examined women aged 40 and over vs. women aged 20–29 and found an increase in the incidence of neonatal asphyxia in older mothers. Notably, the rate of asphyxia was very high: in nulliparous women over the age of 40, it was 6%, vs. 4% in the control group, and in multiparous women, it was 3.4% in older vs. 2.4% in young women. The researchers did not specify how asphyxia was defined, but the high prevalence would hint at a broader definition than ours, which accords with the ICD-9. To the best of our knowledge, the association between AMA and the other severe neonatal outcomes reported here has not previously been examined in the literature.

The present study included only parous women, as nulliparas differ markedly from the parous maternity population in their risks of significantly worse outcomes [16,21,22,23,24,25]. The observed differences between nulliparous and parous women may result from improved placentation [26] due to the decidual natural killer cells of parous women that remember pregnancy. This pregnancy-trained memory dwells in the epigenome (chromatin modification) [27]. Hence, a high percentage of nulliparas included in a cohort shifts the results negatively; for example, Claramonte and colleagues [4] investigated a cohort with 76–93% first births in the various age groups and 72.5% first births in the 40–44 years cohort [4], introducing significant bias. Other previous studies did not report results for nulliparas vs. multiparas. In the current study, we isolated only multiparous women in order to neutralize this pivotal confounder.

### Clinical Implications

Our findings highlight a continuous rise in the rates of CD, PTD, NICU admissions, and several adverse neonatal outcomes with each year increase in maternal age beyond 35 years. Moreover, the increased rate of most of the above-mentioned severe neonatal complications associated with rising maternal age has not previously been reported. Importantly, no clinical inflection point was identified, suggesting a gradual, incremental increase in risk. These results emphasize the need for healthcare providers to counsel women on the potential risks associated with advanced maternal age during family planning discussions. Additionally, these findings have significant implications for professional guidelines and public health planning. Healthcare providers should be aware of these increased risks as they pertain to clinical management and to resource provision planning. These data may be valuable for professional guidelines and public health planning.

This study had several strengths and limitations. The primary strength of this study was our very large cohort, drawn from four large obstetrics departments comprising a catchment area encompassing 25% of the country. This enabled sufficient numbers to stratify risks to successive maternal age increments. Furthermore, our data were retrieved from computerized medical records updated in real time and including each patient’s antepartum, labor and delivery, and postpartum records for each delivery. This reduced the possibility of bias and provided a more complete picture of the complications associated with the history of pregnancy losses. The main limitation of the study was its retrospective nature, with its inherent flaws. A further drawback was that, despite our large cohort, we did not have enough statistical power to analyze rare complications such as maternal mortality and amniotic fluid emboly.

## 5. Conclusions

AMA in multiparous women is associated with an increased rate of complications among mothers and neonates, including CD, PTD, NICU admission, neonatal asphyxia, mechanical ventilation, SGA, TTN, hypoglycemia, and jaundice of the newborn. Rates of these complications rise as a continuum with maternal age, with no clinical inflection point. The increased rate of severe neonatal complications associated with rising maternal age has not previously been reported. These data may be valuable for professional guidelines and public health planning, as these increased risks pertain to clinical management and patient counseling, as well as resource provision planning. Patient family planning counseling should include promoting awareness of the year-on-year increased risk of complications associated with delayed childbearing, replacing the arbitrary cut-off of 35 years. Future prospective studies are needed to examine the relationship between AMA and severe neonatal outcomes.

## Figures and Tables

**Table 1:** Demographic and obstetric characteristics of the study groups.

	Age 25–30 n = 114,918 (Reference Group)	Age 35–36 n = 27,089	*p* Value	Age 37–38 n = 20,450	*p* Value	Age 39–40 n = 13,928	*p* Value	Age 41–42 n = 7802	*p* Value	Age 43–44 n = 3396	*p* Value	Age 45–46 n = 1031	*p* Value
Gravidity	3.8 ± 1.5	6.1 ± 2.7	<0.01	6.9 ± 3.1	<0.01	7.8 ± 3.4	<0.01	8.7 ± 3.8	<0.01	9.8 ± 4.2	<0.01	10.7 ± 4.6	<0.01
Parity	3.4 ± 1.2	5.3 ± 2.3	<0.01	5.9 ± 2.7	<0.01	6.6 ± 3	<0.01	7.4 ± 3.4	<0.01	8.2 ± 3.7	<0.01	8.8 ± 4	<0.01
Previous miscarriages	0.4 ± 0.8	0.9 ± 1.2	<0.01	1 ± 1.3	<0.01	1.2 ± 1.4	<0.01	1.4 ± 1.5	<0.01	1.7 ± 1.7	<0.01	1.9 ± 1.8	<0.01
Number of previous CDs	0.2 ± 0.5	0.3 ± 0.7	<0.01	0.4 ± 0.8	<0.01	0.4 ± 0.9	<0.01	0.4 ± 0.9	<0.01	0.4 ± 0.9	<0.01	0.4 ± 0.9	<0.01
Previous cesarean delivery, any	13,893 (12.8%)	5659 (20.9%)	<0.01	4598 (22.5%)	<0.01	3352 (24.1%)	<0.01	2023 (25.9%)	<0.01	939 (27.7%)	<0.01	278 (27%)	<0.01
Fertility Treatments	1817 (1.7%)	814 (3%)	<0.01	677 (3.3%)	<0.01	521 (3.7%)	<0.01	335 (4.3%)	<0.01	160 (4.7%)	<0.01	74 (7.2%)	<0.01
Hypertensive disorders of pregnancy	1097 (1%)	532 (2%)	<0.01	531 (2.6%)	<0.01	410 (2.9%)	<0.01	310 (4%)	<0.01	168 (4.9%)	<0.01	64 (6.2%)	<0.01
Diabetes (pre-gestational and gestational)	2178 (2.1%)	1297 (5.1%)	<0.01	1230 (6.4%)	<0.01	995 (7.6%)	<0.01	729 (9.9%)	<0.01	363 (11.4%)	<0.01	110 (11.4%)	<0.01
Obesity (BMI > 30)	2967 (11.1%)	1182 (16.4%)	<0.01	980 (16.9%)	<0.01	758 (18.3%)	<0.01	454 (18.4%)	<0.01	216 (19%)	<0.01	61 (17.5%)	<0.01
Smoking	2139 (2.2%)	795 (3.2%)	<0.01	586 (3.2%)	<0.01	393 (3.1%)	<0.01	207 (2.9%)	<0.01	91 (3%)	0.01	30 (3.2%)	0.03
Hemoglobin at admission to labor <11gr%	8863 (11.3%)	2234 (12.4%)	<0.01	1582 (11.4%)	0.87	1167 (12.2%)	0.01	583 (10.4%)	0.04	244 (9.8%)	0.02	80 (10.4%)	0.45
Induction of labor	9412 (8.8%)	3223 (12.2%)	<0.01	2496 (12.6%)	<0.01	1796 (13.4%)	<0.01	970 (13%)	<0.01	462 (14.3%)	<0.01	146 (15%)	<0.01
Oxytocin augmentation of labor	44,462 (41%)	10,737 (39.6%)	<0.01	8258 (40.4%)	0.08	5612 (40.3%)	0.09	3265 (41.8%)	0.16	1377 (40.5%)	0.57	443 (43%)	0.21
Epidural analgesia	48,965 (45.2%)	12,393 (45.7%)	0.10	9023 (44.1%)	<0.01	5933 (42.6%)	<0.01	3175 (40.7%)	<0.01	1315 (38.7%)	<0.01	366 (35.5%)	<0.01

Data are mean ± standard deviation or number (%). *p* values were calculated from Chi-square or *t*-test, as appropriate, while comparing each stratum to the age group 25–30 years old. BMI—body mass index, CD—cesarean delivery.

**Table 2:** Obstetric and maternal outcomes among the study groups.

	Age 25–30 n = 114,918 (Reference Group)	Age 35–36 n = 27,089	*p* Value	Age 37–38 n = 20,450	*p* Value	Age 39–40 n = 13,928	*p* Value	Age 41–42 n = 7802	*p* Value	Age 43–44 n = 3396	*p* Value	Age 45–46 n = 1031	*p* Value
Preterm:													
Gestational age at delivery ≤ 37 week	3506 (3.2%)	1138 (4.2%)	<0.01	916 (4.5%)	<0.01	676 (4.9%)	<0.01	383 (4.9%)	<0.01	181 (5.3%)	<0.01	63 (6.1%)	<0.01
Gestational age at delivery ≤ 34 week	579 (0.5%)	170 (0.6%)	0.06	148 (0.7%)	<0.01	115 (0.8%)	<0.01	66 (0.8%)	<0.01	66 (0.8%)	0.37	14 (1.4%)	<0.01
Gestational age at delivery ≤ 32 week	290 (0.3%)	78 (0.3%)	0.57	74 (0.4%)	0.02	57 (0.4%)	<0.01	36 (0.5%)	<0.01	36 (0.5%)	0.53	6 (0.6%)	0.05
Gestational age at delivery ≤ 28 week	75 (0.1%)	25 (0.1%)	0.21	19 (0.1%)	0.25	17 (0.1%)	0.03	12 (0.2%)	<0.01	12 (0.2%)	0.29	0 (0%)	0.40
Mode of delivery:													
Unplanned cesarean	2475 (2.3%)	941 (3.5%)	<0.01	782 (3.8%)	<0.01	636 (4.6%)	<0.01	390 (5%)	<0.01	187 (5.5%)	<0.01	80 (7.8%)	<0.01
Cesarean delivery	7296 (6.7%)	3665 (13.5%)	<0.01	3179 (15.5%)	<0.01	2503 (18%)	<0.01	1555 (19.9%)	<0.01	764 (22.5%)	<0.01	279 (27.1%)	<0.01
Vacuum assisted delivery	2697 (2.5%)	763 (2.8%)	<0.01	537 (2.6%)	0.25	375 (2.7%)	0.15	226 (2.9%)	0.03	95 (2.8%)	0.26	30 (2.9%)	0.39
Obstetric outcomes:													
Placental abruption	1333 (1.3%)	438 (1.7%)	<0.01	366 (1.9%)	<0.01	241 (1.8%)	<0.01	175 (2.3%)	<0.01	62 (1.9%)	<0.01	27 (2.7%)	<0.01
Episiotomy	3288 (3%)	572 (2.1%)	<0.01	394 (1.9%)	<0.01	205 (1.5%)	<0.01	105 (1.3%)	<0.01	41 (1.2%)	<0.01	16 (1.6%)	0.01
Perineal tear grade 3/4	162 (0.1%)	9 (0%)	<0.01	8 (0%)	<0.01	7 (0.1%)	<0.01	3 (0%)	0.01	0 (0%)	0.02	0 (0%)	0.21
Shoulder dystocia	306 (0.4%)	83 (0.5%)	0.18	57 (0.4%)	0.74	54 (0.6%)	0.01	25 (0.4%)	0.52	16 (0.6%)	0.05	2 (0.3%)	0.57
Meconium-stained amniotic fluid	16,005 (14.8%)	4476 (16.5%)	<0.01	3382 (16.5%)	<0.01	2332 (16.7%)	<0.01	1329 (17%)	<0.01	630 (18.6%)	<0.01	188 (18.2%)	<0.01
Postpartum hemorrhage	4497 (4.3%)	1127 (4.4%)	0.87	906 (4.6%)	0.08	615 (4.6%)	0.12	382 (5.1%)	<0.01	191 (5.9%)	<0.01	64 (6.5%)	<0.01
Chorioamnionitis	566 (0.5%)	146 (0.5%)	0.74	97 (0.5%)	0.38	77 (0.6%)	0.64	41 (0.5%)	0.97	18 (0.5%)	0.95	10 (1%)	0.05
Puerperal fever	655 (0.8%)	232 (1.3%)	<0.01	172 (1.2%)	<0.01	132 (1.4%)	<0.01	85 (1.5%)	<0.01	41 (1.6%)	<0.01	9 (1.2%)	0.31
Blood product transfusion	512 (0.7%)	201 (1.1%)	<0.01	130 (0.9%)	<0.01	93 (1%)	<0.01	69 (1.2%)	<0.01	30 (1.2%)	<0.01	11 (1.4%)	0.01
Hysterectomy	11 (0%)	22 (0.1%)	<0.01	15 (0.1%)	<0.01	18 (0.2%)	<0.01	7 (0.1%)	<0.01	5 (0.2%)	<0.01	1 (0.1%)	0.01
Uterine rupture	69 (0.1%)	15 (0.1%)	0.62	16 (0.1%)	0.46	13 (0.1%)	0.20	1 (0%)	0.08	2 (0.1%)	0.91	0 (0%)	0.42
Laparotomy	19 (0%)	7 (0%)	0.28	11 (0.1%)	<0.01	6 (0.1%)	0.04	2 (0%)	0.60	5 (0.2%)	<0.01	0 (0%)	0.67
Maternal ICU admissions	22 (0%)	16 (0.1%)	<0.01	3 (0%)	0.67	6 (0.1%)	0.07	5 (0.1%)	0.01	2 (0.1%)	0.14	1 (0.1%)	0.10
Prolonged hospital stays	559 (0.7%)	270 (1.5%)	<0.01	225 (1.6%)	<0.01	166 (1.7%)	<0.01	110 (2%)	<0.01	64 (2.6%)	<0.01	18 (2.3%)	<0.01

Data are mean ± standard deviation or number (%). *p* values were calculated from Chi-square or *t*-test, as appropriate, while comparing each stratum to the age group 25–30 years old. ICU—intensive care unit.

**Table 3:** Neonatal outcomes among the study groups.

	Age 25–30 n = 114,918 (Reference Group)	Age 35–36 n = 27,089	*p* Value	Age 37–38 n = 20,450	*p* Value	Age 39–40 n = 13,928	*p* Value	Age 41–42 n = 7802	*p* Value	Age 43–44 n = 3396	*p* Value	Age 45–46 n = 1031	*p* Value
Neonatal characteristics:													
Birth weight	3329 ± 454.6	3356.2 ± 480	<0.01	3358.3 ± 494.3	<0.01	3363.3 ± 498.8	<0.01	3375.7 ± 507.3	<0.01	3358.2 ± 519.3	<0.01	3341.6 ± 519.2	0.38
Macrosomia (birth weight ≥ 4000 g)	6061 (5.6%)	1853 (6.8%)	<0.01	1452 (7.1%)	<0.01	1054 (7.6%)	<0.01	578 (7.4%)	<0.01	236 (7%)	<0.01	65 (6.3%)	0.32
LGA	12,069 (11.1%)	3403 (12.6%)	<0.01	2702 (13.2%)	<0.01	1913 (13.7%)	<0.01	1143 (14.7%)	<0.01	491 (14.5%)	<0.01	122 (11.8%)	0.49
SGA	8379 (7.7%)	2337 (8.6%)	<0.01	1765 (8.6%)	<0.01	1246 (9%)	<0.01	733 (9.4%)	<0.01	326 (9.6%)	<0.01	100 (9.7%)	0.02
Male gender	55,941 (51.6%)	14004 (51.7%)	0.87	10,527 (51.5%)	0.67	7029 (50.5%)	0.01	3992 (51.2%)	0.43	1756 (51.7%)	0.93	541 (52.5%)	0.60
Neonatal outcomes:													
5-Minute Apgar score < 7	933 (0.9%)	269 (1%)	0.04	245 (1.2%)	<0.01	140 (1%)	0.09	98 (1.3%)	<0.01	56 (1.7%)	<0.01	14 (1.4%)	0.09
NICU admission	2909 (2.7%)	893 (3.3%)	<0.01	724 (3.5%)	<0.01	555 (4%)	<0.01	314 (4%)	<0.01	187 (5.5%)	<0.01	62 (6%)	<0.01
Encephalopathy	33 (0%)	9 (0%)	0.82	6 (0%)	0.93	4 (0%)	0.91	5 (0.1%)	0.11	2 (0.1%)	0.36	1 (0.1%)	0.23
Intracranial hemorrhage	129 (0.1%)	52 (0.2%)	<0.01	41 (0.2%)	<0.01	32 (0.2%)	<0.01	17 (0.2%)	0.02	5 (0.1%)	0.64	0 (0%)	0.27
Birth asphyxia	361 (0.3%)	83 (0.3%)	0.49	97 (0.5%)	<0.01	72 (0.5%)	<0.01	50 (0.6%)	<0.01	22 (0.6%)	<0.01	10 (1%)	<0.01
Mechanical ventilation	737 (0.7%)	211 (0.8%)	0.08	181 (0.9%)	<0.01	145 (1%)	<0.01	80 (1%)	<0.01	44 (1.3%)	<0.01	19 (1.8%)	<0.01
Seizures	710 (0.7%)	180 (0.7%)	0.87	144 (0.7%)	0.43	126 (0.9%)	<0.01	71 (0.9%)	0.01	37 (1.1%)	<0.01	10 (1%)	0.21
Sepsis	399 (0.4%)	112 (0.4%)	0.28	77 (0.4%)	0.86	49 (0.4%)	0.76	36 (0.5%)	0.19	14 (0.4%)	0.68	6 (0.6%)	0.26
Meconium aspiration syndrome	111 (0.1%)	34 (0.1%)	0.30	24 (0.1%)	0.55	18 (0.1%)	0.36	13 (0.2%)	0.09	6 (0.2%)	0.19	1 (0.1%)	0.96
Jaundice	5554 (5.1%)	1629 (6%)	<0.01	1298 (6.3%)	<0.01	935 (6.7%)	<0.01	512 (6.6%)	<0.01	236 (7%)	<0.01	81 (7.9%)	<0.01
TTN	1391 (1.3%)	411 (1.5%)	<0.01	342 (1.7%)	<0.01	241 (1.7%)	<0.01	155 (2%)	<0.01	71 (2.1%)	<0.01	22 (2.1%)	0.02
Erb’s palsy/fracture of clavicle	663 (0.6%)	182 (0.7%)	0.26	141 (0.7%)	0.20	82 (0.6%)	0.74	61 (0.8%)	0.07	28 (0.8%)	0.12	5 (0.5%)	0.60
Hypoglycemia	3068 (2.8%)	1056 (3.9%)	<0.01	961 (4.7%)	<0.01	751 (5.4%)	<0.01	498 (6.4%)	<0.01	237 (7%)	<0.01	82 (8%)	<0.01

LGA—large for gestational age, SGA—small for gestational age, NICU—neonatal intensive care unit, TTN—transient tachypnea of the newborn.

**Table 4:** Multinomial multivariate logistic regression analysis for the association between maternal age and mode of delivery, preterm delivery, and NICU admission (adjusted odds ratio).

		Adjusted Odds Ratios (95% CI) *						
		Age 35–36	Age 37–38	Age 39–40	Age 41–42	Age 43–44	Age 45–46	Per Unit Increase **
Mode of delivery ***	Vacuum	1.8 (1.67–2.01)	2.0 (1.77–2.19)	2.5 (2.18–2.8)	3.2 (2.72–3.73)	3.8 (2.99–4.76)	5.3 (3.58–7.93)	1.06 (1.05–1.08)
	Elective cesarean	2.3 (2.1–2.46)	3.1 (2.86–3.4)	4.3 (3.87–4.72)	5.7 (5.1–6.48)	8.1 (6.9–9.58)	13.4 (10.21–17.52)	1.12 (1.11–1.13)
	Unplanned cesarean	2.0 (1.84–2.15)	2.3 (2.08–2.48)	3.1 (2.85–3.47)	3.7 (3.26–4.16)	5.0 (4.24–5.89)	8.0 (6.19–10.42)	1.07 (1.06–1.09)
Preterm delivery < 37		1.2 (1.00–1.42)	1.4 (1.15–1.68)	1.5 (1.18–1.8)	1.3 (0.97–1.68)	1.3 (0.9–1.89)	1.7 (0.93–2.93)	1.02 (1.01–1.04)
NICU admission		1.2 (1.01–1.38)	1.1 (0.92–1.33)	1.3 (1.1–1.64)	1.1 (0.88–1.46)	1.7 (1.23–2.27)	1.8 (1.11–2.95)	1.02 (1.01–1.04)
Composite adverse neonatal outcomes ****		1.1 (0.99–1.2)	1.2 (1.1–1.36)	1.4 (1.23–1.56)	1.2 (1.06–1.44)	1.4 (1.13–1.68)	1.5 (1.07–2.05)	1.02 (1.02–1.03)

* Adjusted for number of previous cesarean deliveries, hypertensive disorders of pregnancy, smoking, fertility treatments, diabetes, previous miscarriages, obesity (BMI > 30), gravidity, parity, and year; age 25–30 constituted the reference group; ** maternal age was treated as a continuous variable; *** spontaneous vaginal delivery consisting the reference group. NICU—neonatal intensive care unit. **** Composite adverse neonatal outcomes include at least one of the following: meconium aspiration syndrome, jaundice, TTN, mechanical ventilation, seizures, Erb’s palsy/fracture of clavicle, hypoglycemia, sepsis, encephalopathy, and intracranial hemorrhage.

## Data Availability

Due to the privacy concerns surrounding patient data, the data are not available for sharing.

## Supplementary Materials

The following supporting information can be downloaded at: https://www.mdpi.com/article/10.3390/jcm12175696/s1, Figure S1: Flow chart of the study group.

## References

[1] Martin J.A., Hamilton B.E., Ventura S.J., Menacker F., Park M.M., Sutton P.D. (2002). Births: Final data for 2001. Natl. Vital Stat. Rep..

[2] Osterman M.J., Hamilton B.E., Martin J.A., Driscoll A.K., Valenzuela C.P. (2022). Births: Final Data for 2020.

[3] Yogev Y., Melamed N., Bardin R., Tenenbaum-Gavish K., Ben-Shitrit G., Ben-Haroush A. (2010). Pregnancy outcome at extremely advanced maternal age. Am. J. Obstet. Gynecol..

[4] Claramonte Nieto M., Meler Barrabes E., Garcia Martinez S., Gutierrez Prat M., Serra Zantop B. (2019). Impact of aging on obstetric outcomes: Defining advanced maternal age in Barcelona. BMC Pregnancy Childbirth.

[5] Osmundson S.S., Gould J.B., Butwick A.J., Yeaton-Massey A., El-Sayed Y.Y. (2016). Labor outcome at extremely advanced maternal age. Am. J. Obstet. Gynecol..

[6] Kahveci B., Melekoglu R., Evruke I.C., Cetin C. (2018). The effect of advanced maternal age on perinatal outcomes in nulliparous singleton pregnancies. BMC Pregnancy Childbirth.

[7] Fuchs F., Monet B., Ducruet T., Chaillet N., Audibert F. (2018). Effect of maternal age on the risk of preterm birth: A large cohort study. PLoS ONE.

[8] Lisonkova S., Potts J., Muraca G.M., Razaz N., Sabr Y., Chan W.-S., Kramer M.S. (2017). Maternal age and severe maternal morbidity: A population-based retrospective cohort study. PLoS Med..

[9] Waldenstrom U., Cnattingius S., Vixner L., Norman M. (2017). Advanced maternal age increases the risk of very preterm birth, irrespective of parity: A population-based register study. BJOG.

[10] Khalil A., Syngelaki A., Maiz N., Zinevich Y., Nicolaides K.H. (2013). Maternal age and adverse pregnancy outcome: A cohort study. Ultrasound Obstet. Gynecol..

[11] Sheen J.J., Wright J.D., Goffman D., Kern-Goldberger A.R., Booker W., Siddiq Z., D’alton M.E., Friedman A.M. (2018). Maternal age and risk for adverse outcomes. Am. J. Obstet. Gynecol..

[12] Higgins R.D., Saade G., Polin R.A., Grobman W.A., Buhimschi I.A., Watterberg K., Silver R.M., Raju T.N. (2016). Evaluation and Management of Women and Newborns with a Maternal Diagnosis of Chorioamnionitis: Summary of a Workshop. Obstet. Gynecol..

[13] Newton E.R. (2005). Preterm labor, preterm premature rupture of membranes, and chorioamnionitis. Clin. Perinatol..

[14] Hollander D. (1986). Diagnosis of chorioamnionitis. Clin. Obstet. Gynecol..

[15] Gibbs R.S. (1977). Diagnosis of intra-amniotic infection. Semin. Perinatol..

[16] Gabbe S., Niebyl J., Simpson J., Landon M., Galan H., Kilpatrick S., Garrison E. (2012). Normal labor and delivery. Obstetrics: Normal and Problem Pregnancies.

[17] Dollberg S., Haklai Z., Mimouni F.B., Gorfein I., Gordon E.S. (2005). Birth weight standards in the live-born population in Israel. Isr. Med. Assoc. J..

[18] Jacobs S.E., Berg M., Hunt R., Tarnow-Mordi W.O., Inder T.E., Davis P.G. (2013). Cooling for newborns with hypoxic ischaemic encephalopathy. Cochrane Database Syst. Rev..

[19] Bayrampour H., Heaman M. (2010). Advanced maternal age and the risk of cesarean birth: A systematic review. Birth.

[20] Gilbert W.M., Nesbitt T.S., Danielsen B. (1999). Childbearing beyond age 40: Pregnancy outcome in 24,032 cases. Obstet. Gynecol..

[21] Ballering G., Leijnse J., Eijkelkamp N., Peeters L., de Heus R. (2018). First-trimester placental vascular development in multiparous women differs from that in nulliparous women. J. Matern. Fetal Neonatal Med..

[22] Stillbirth Collaborative Research Network Writing Group (2011). Association between stillbirth and risk factors known at pregnancy confirmation. JAMA.

[23] Brosens I., Pijnenborg R., Vercruysse L., Romero R. (2011). The “Great Obstetrical Syndromes” are associated with disorders of deep placentation. Am. J. Obstet. Gynecol..

[24] Shah P.S., Knowledge Synthesis Group on Determinants of LBW/PT births (2010). Parity and low birth weight and preterm birth: A systematic review and meta-analyses. Acta Obstet. Gynecol. Scand..

[25] Clapp J.F., Capeless E. (1997). Cardiovascular function before, during, and after the first and subsequent pregnancies. Am. J. Cardiol..

[26] Gamliel M., Goldman-Wohl D., Isaacson B., Gur C., Stein N., Yamin R., Berger M., Grunewald M., Keshet E., Rais Y. (2018). Trained Memory of Human Uterine NK Cells Enhances Their Function in Subsequent Pregnancies. Immunity.

[27] Goldman-Wohl D., Gamliel M., Mandelboim O., Yagel S. (2019). Learning from experience: Cellular and molecular bases for improved outcome in subsequent pregnancies. Am. J. Obstet. Gynecol..

