# Supplementary figures and images for: The Impact of Advanced Maternal Age on Pregnancy Outcomes: A Retrospective Multicenter Study
Source: J Clin Med. 2023 Sep 1;12(17):5696. doi: 10.3390/jcm12175696 (PMC10488955; doi:10.3390/jcm12175696)

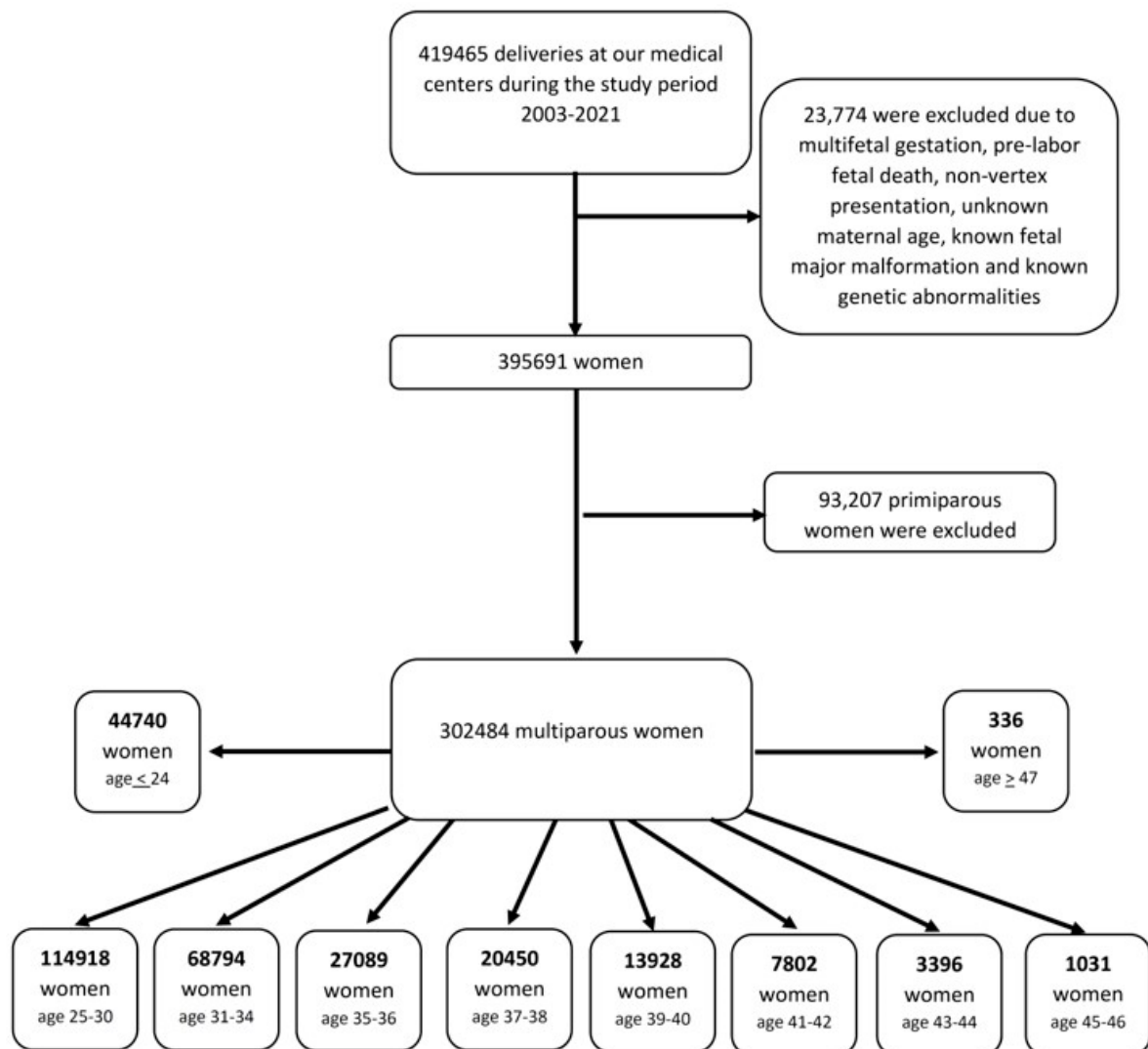

**Figure S1.** Flow chart of the study group.

Supplement: Supplementary file 1 [file jcm-12-05696-s001.zip › jcm-2542328-supplementary.pdf]
